# 

**Published:** 1877-06

**Authors:** 


### PENNSYLVANIA STATE DENTAL SOCIETY.

The ninth Annual Meeting of the Pennsylvania State Dental Society will convene at Minnequa Springs, Tuesday, July 10th, at 10 o'clock A.M.

A full list of essayists and subjects will be announced in the next number of this journal. It is designed to have surgical as well as operative clinics. Dentists having cases suitable for surgical clinics will confer a favor by corresponding with the executive committee at once.

Minnequa is 40 miles north of Williamsport, on the Allegheny range, a delightfully cool and pleasant summer resort.

The hotel rates to persons attending the Society will not exceed $2.00 per day. Excursion tickets can be obtained at all principal points.

Ample arrangements are being made for the comfort and accommodation of all who will attend.

Rooms can be secured by addressing W. D. Tyler, Sup't., Minnequa, Pa., or the undersigned. All members of the Dental profession from this and other states are invited to be present.

G. W. KLUMP,

*Chairman Executive Committee, Williamsport, Pa.*

### INDIANA STATE DENTAL ASSOCIATION.

The nineteenth Annual Meeting of the Indiana State Dental Association, will be held in Armory Hall, Fort Wayne, commencing Tuesday, June 26th, 1877, 10 o'clock A.M.

REPORTS OF SPECIAL COMMITTEES.

Dental Legislation, M. H. Herriott, J. E. Cravens; Conservative Dentistry, J. R. Clayton, S. T. Kirk; Exposed Pulp, J. E. Cravens, M. Wells, F. C. Eddleman; Treatment of Pulpless Teeth, and Alveolar Abcess, J. A. Turner, J. W. Ellis, S. C. Carter; Care of Children's Teeth, G. W. Keely, S. M. Goode; Tin and Amalgam for Filling Teeth, J. K. Jameson, S. B. Brown; Dental Literature, J. W. Jay; Dental Ethics, M. H. Chappell, W. E. Driscoll, O. A. Beeks.

SUBJECTS FOR DISCUSSION.

Microscopy, Replantation of Teeth, Treatment of Sensitive Dentine.

Dr. Cushing, of Chicago has *positively* promised to attend the full sessions of the association.

Drs. Taft, Keely and Smith, have given strong assurances of their purpose to be present, and are confidently expected.

The Fort Wayne, Muncie & Cincinnati Rail Road, will *return* members *free*, from Fort Wayne to Connersville and intermediate stations. The Wabash Rail Road Company, will sell round trip tickets at a discount of about 25 per cent.

HOTEL RATES.

Aveline House, $2,00 per day; Mayer House, $1,50 per day.

### DEATH FROM CHLOROFORM.

Death of Dr. Charles A. Jourdan, from the direct effects of Chloroform:—

At half past four o'clock P. M. Dr. Jourdan was put under the influence of cloroform, for the purpose of having the left eye extirpated, which had been injured five months peviously.

The patient was placed upon the operating table and chloroform administered in the usual way, which soon brought on that sleep that knows no wakening. The eye was rapidly removed, when the pulse and breath simultaneously ceased, and all efforts to resusitate were unavailing; he did not breathe after the first alarming symtoms were noticed.

He was forty-seven years of age, stout and robust and no organic disease of any kind, which was proved by post-mortem examination forty-eight hours after death.

Dr. Jourdan was born and raised in East Tenn., and practiced dentistry many years in Athens, Tenn. For the last ten years, he practiced in Huntsville, Ala., until twelve months ago when he removed to Lovell, Texas, where he practiced until he received the accident above named.

Dr. Jourdan was a true gentleman of the old school, and an *honest man.*

As an operator he was among the first, as a friend true and noble: to know him was to like him—society and the profession have sustained a great loss, and his family an irreparable bereavement.

Memphis, Tenn., April 7th, 1877. S. P. CUTLER.

### TENNESSEE DENTAL SOCIETY.

The Annual Meeting of the Tennessee State Dental Society, will be held at Lookout Mountain, commencing Wednesday morning at 10 o'clock June 27th. Reduced Railroad and Hotel rates have been secured. All are invited, a large attendance is expected, let all come prepared to make it a grand meeting

### SOUTHERN DENTAL ASSOCIATION.

In accordance with a resolution passed the last Annual Meeting of "the Southern Dental Association," held at Montgomery, Ala., requesting the Presiding Officer, to call a special session ; I hereby notify all members of the above-mentioned society, that there will be a Special Session held at Deer Park, Md., commencing Tuesday, August 14th, 1877, for the purpose of participating in a joint meeting of "the American Dental Convention," —"Maryland and District of Columbia Association."

Drs. R. Finley Hunt, of Washington; R. B. Winder, of Baltimore, and J. R. Walker, of New Orleans, having been appointed a Commitee of Arrangement for this Special Session, it is to be hoped, that they will leave nothing undone, that will add to the interest of the meeting.

S. J. Cobb, *Pres't.*

E. S. Chisholm, *Rec. Sec'y.*

### THE SEMI-ANNUAL MEETING OF THE CONNECTICUT VALLEY DENTAL SOCIETY FOR 1877.

This meeting will be held at the Mansion House, Greenfield, Mass., on Tuesday and Wednesday, June 12th and 13th, commencing on Tuesday at 10 A. M.

SUBJECTS AND ESSAYISTS.

1. Mechanical Dentistry. Dr. C. C. Haskell, Greenfield, Mass.

2. Dental Education and the Obligations of the Practitioner to his Student. Dr. C. Johnson, Thompsonville, Conn.

3. Incidents of Practice, Special Operations, etc. Dr. D. H. Smith, Holyoke, Mass.

4. What have the public a right to expect from the Dental Practitioner? Dr. J. J. Vincent, Amherst, Mass.

5. Filling Teeth, and Preparations of Gold for Filling. Dr. L. C. Taylor, Hartford, Conn.

6. Dental Appliances, Pet Instruments, Clinic with Non-cohesive Foil, etc. Dr. L. D. Shepard, Boston, Mass. (and others.)

7. Plastic Fillings. Dr. C. L. Anderson, Springfield, Mass.

8. Miscellaneous Subjects.

The Executive Committee propose that the time from 8 to 10 A. M. on Wednesday be set apart for the special consideration of the sixth subject on the programme. It is the intention to provide two or more chairs, at which several operations may be in progress at the same time, if desired, affording an opportunity for different members to illustrate their own methods of performing some simple operation, such as applying the Rubber Dam, introducing a Temporary Filling, etc.

Prof. L. D. Shepard, of Boston, will give a clinic illustrating the making and use of Cylinders of Non-cohesive Foil, put in by Hand Pressure and Automatic Mallet. The Exhibition of New Appliances and Pet Instruments will also be in order at this time. Accommodations at the Mansion House may be had for $2.50 per day. An excursion to the "Tunnel" after adjournment has been suggested, and will be submitted to the Society.

Members of the profession generally are cordially invited to be present. C. T. Stockwell, Secretary.

Springfield, Mass., April 20th, 1877,

### CONTENTS.

### COMMUNICATIONS.

A MONTHLY MAGAZINE DEVOTED TO THE INTERESTS OF

**THE DENTAL PROFESSION.**

*TERMS REDUCED TO $2.50 PER YEAR. SINGLE COPIES 25C.*

EDITED BY PROF. J. TAFT, D.D.S.

AND

*Published by SPENCER, CROCKER & CO., Ohio Dental Depot.*

The volume commences in January and closes in December.

The REGISTER being issued monthly is always fresh, therefore Indispensable to the practitioner who desires to keep posted up with the new inventions and improvements which are daily developed. Neither expense nor effort will be spared to give value and variety to its contents. Articles will be illustrated with well executed engravings whenever they will add to the interest or assist to explanation.

Its extensive circulation and frequency of issue make it one of the BEST DENTAL ADVERTISING MEDIUMS in the United States.

All subscriptions must begin with January or July.

Local or special notices, five lines or less, will be inserted for $3 each insertion; over five lines, 50 cts. per line.

All advertising bills payable in advance, or at furthest, after the issue of the first number containing the advertisement. All transient advertisements to be paid for in advance.

☞CONTRIBUTIONS SOLICITED.

Exclusively Editorial Communications should be addressed to Editor.

The latest number of the REGISTER may be ordered with or without other goods at any time, and all letters should be addressed to

***SPENCER, CROCKER & CO., Ohio Dental Depot, Cincinnati, O.***

